# High-Intensity Focused Ultrasound Circular Cyclocoagulation in Glaucoma: A Step Forward for Cyclodestruction?

**DOI:** 10.1155/2017/7136275

**Published:** 2017-04-22

**Authors:** Rodolfo Mastropasqua, Vincenzo Fasanella, Alessandra Mastropasqua, Marco Ciancaglini, Luca Agnifili

**Affiliations:** ^1^Moorfields Eye Hospital, London EC1V 2PD, UK; ^2^Ophthalmology Clinic, Department of Medicine and Ageing Science, University G. d'Annunzio of Chieti-Pescara, 66100 Chieti, Italy; ^3^Ophthalmology Unit, Department of Life, Health and Environmental Sciences, University of L'Aquila, 67100 L'Aquila, Italy

## Abstract

The ciliary body ablation is still considered as a last resort treatment to reduce the intraocular pressure (IOP) in uncontrolled glaucoma. Several ablation techniques have been proposed over the years, all presenting a high rate of complications, nonselectivity for the target organ, and unpredictable dose-effect relationship. These drawbacks limited the application of cyclodestructive procedures almost exclusively to refractory glaucoma. High-intensity focused ultrasound (HIFU), proposed in the early 1980s and later abandoned because of the complexity and side effects of the procedure, was recently reconsidered in a new approach to destroy the ciliary body. Ultrasound circular cyclocoagulation (UC^3^), by using miniaturized transducers embedded in a dedicated circular-shaped device, permits to selectively treat the ciliary body in a one-step, computer-assisted, and non-operator-dependent procedure. UC^3^ shows a high level of safety along with a predictable and sustained IOP reduction in patients with refractory glaucoma. Because of this, the indication of UC^3^ was recently extended also to naïve-to-surgery patients, thus reconsidering the role and timing of ciliary body ablation in the surgical management of glaucoma. This article provides a review of the most used cycloablative techniques with particular attention to UC^3^, summarizing the current knowledge about this procedure and future possible developments.

## 1. Introduction

Lowering intraocular pressure (IOP) is the only proven approach to reduce the rate of retinal ganglion cell loss and the rate of progression in patients with glaucoma [1]. Nevertheless, in many cases, medical and surgical approaches do not reach the required target IOP [2].

Refractory glaucoma comprises all forms of glaucoma in which the IOP remained uncontrolled despite maximum-tolerated medical therapy and previous laser or surgical procedures. In this case, the IOP remains uncontrolled also after repeated standard filtration surgeries. Surgical approaches for refractory glaucoma include techniques increasing the aqueous humor (AH) outflow (filtrating procedures, drainage devices) and techniques reducing the AH inflow, by destroying portions of the ciliary body [3].

Several cycloablative methods have been proposed over the years, such as cryotherapy, microwave heating, endoscopic laser coagulation, and transscleral diode laser photocoagulation, which remain the most used ablative procedure [4–7]. Given the occurrence of potential vision-threatening complications, unpredictable dose-effect relationship, significant variability in the final IOP lowering, and poor reproducibility, the ciliary body ablation is still considered as a last resort treatment, recommended only in patients with refractory glaucoma [8].

The high-intensity focused ultrasound (HIFU) technology was proposed as a safer alternative of ciliary body destruction in the 1980s and 1990s (Therapeutic Ultrasound System; Sonocare Inc., Ridgewood, NJ) [9–15]. This technique permits a selective thermic effect on the target organ, limits damage to neighbouring tissues, and allows treating nonoptically transparent structures [9]. Nevertheless, because of the excessive complexity and duration of the treatment, the technique was progressively abandoned despite evidence of good efficacy and safety [14, 15]. In the last years, HIFU has been reconsidered after critical technical modifications and significant improvements in all steps of the procedure.

The ultrasonic circular cyclocoagulation (UC^3^) is an automated, computer-assisted, non-operator-dependent cycloablative procedure that utilizes a circular-shaped probe matching the three-dimensional anatomy of the ciliary body. The particular geometry of the probe permits correctly focusing the target organ. Recent studies in patients with refractory glaucoma report encouraging results after UC^3^ in terms of both IOP reduction and safety of the procedure [16–21].

This article provides a review of all cycloablative techniques proposed over the years, giving particular attention to mechanisms of action, efficacy, safety, and possible future developments of UC^3^.

## 2. Methods

PubMed searches were performed on June 20, 2016, using the following phrases: cyclo-ablation and refractory glaucoma or open angle glaucoma; cyclo-destruction and refractory glaucoma or open angle glaucoma; high-intensity focused ultrasound cyclo-ablation and refractory glaucoma or open angle glaucoma; ultrasonic circular cyclo-coagulation and refractory glaucoma or open angle glaucoma. The searches identified 86 unique publications in English, which were considered for the present review. Publications that were not in English were included only if they provided enough information in the English abstract. All studies considered in the present review met the following inclusion criteria: patients get and signed an informed consent after explanation of the nature and possible consequences of the study and were approved by an Ethics Committee and/or Institutional Review Board.

## 3. Cyclodestructive Techniques

Several cyclodestructive procedures have been proposed during the past 70 years.

The first report was the surgical excision of the ciliary body, named as cyclectomy, which required a full thickness scleral flap to expose the ciliary body, with a following up to one-quarter removal of the organ [22]. Despite a substantial good efficacy, the procedure was rapidly abandoned because of serious complications such as phthisis bulbi and vitreous and expulsive hemorrhages [23].

The following procedures proposed to ablate the ciliary body avoided the excision and aimed at ablate-selected portions of the ciliary processes, by using different physical approaches. Generally, the epithelial cell destruction is considered as the major mechanism for the reduction of the AH secretion and, thus, the IOP.

In cyclodiathermy, heat was transsclerally delivered by using a round-tipped probe attached to a cautery unit in order to destroy selective portions of the ciliary body epithelium [24]. Vogt subsequently modified the technique proposing the penetrating cyclodiathermy; in this procedure, the probe penetrates the sclera and directly treats the ciliary body [25]. Initial reports were encouraging, but studies with longer follow-up produced very poor results since only the 5% of the treated eyes presented a well-controlled IOP [26]. Moreover, serious postoperative complications similar to those described in cyclectomy were frequently described [27]. Therefore, with the diffusion of newer and safer cryodestructive techniques, the use of cyclodiathermy was progressively abandoned.

Cyclocryotherapy allowed treating the ciliary body in a less destructive and more predictable way than cyclodiathermy, exploiting the effects of freezing. This approach was found to reduce the mean IOP from 7.9 to 24.3 mmHg in refractory open- or closed-angle glaucoma [28]. The IOP was better controlled in angle closure or primary open-angle glaucoma (POAG) (66.7% of cases) compared to that in secondary open-angle glaucoma (0%), with the success rate ranging from 57% to 76% [4, 29].

Despite good efficacy, cyclocryotherapy presents several postoperative complications, both mild (pain, anterior chamber inflammation, or a transient hyphema in neovascular glaucoma) and severe or vision threatening (persistent hypotony, choroidal detachment, visual acuity loss, and phthisis bulbi) (Table 1). The significant risk for vision-threatening adverse events limits the spectrum of application of this procedure, except for neovascular glaucoma where it is still considered as a valid therapeutic option [3, 28, 30–42].

In other cases, the use of cyclocryotherapy is indicated in end-stage glaucoma and in patients with a poor visual acuity, because of the high risk of visual loss [29].

To date, cyclophotocoagulation still represents the most widely used cycloablative procedure. The transpupillary cyclocoagulation, which utilizes argon laser, has the advantage to directly treat the ciliary body without the need to pass through the sclera. However, the procedure presents a poor efficacy in terms of IOP reduction [42–44]. The transscleral cyclophotocoagulation uses lasers with shorter wavelengths, with the neodymium-yttrium aluminum garnet (Nd:YAG) being the most diffuse. This kind of laser allows penetrating the sclera more effectively and with less back scatter than other kinds of short-wavelength lasers [45]. Histopathology studies showed atrophy of the ciliary processes 1-2 months after Nd:YAG cyclophotocoagulation, with ablations of the secretive epithelium and vasculature necrosis, leading to significant IOP lowering [46–48]. Several studies documented a good efficacy of this technique in reducing the IOP in patients with refractive glaucoma [49–51].

Nd:YAG cyclophotocoagulation can be performed in a noncontact or a contact way. However, though noncontact Nd:YAG cyclophotocoagulation showed encouraging results, the high rate of complications related to the procedure (anterior chamber inflammation, choroidal detachment, transient hypotony, sympathetic ophthalmia, and scleral thinning) led the transscleral contact approach to become the most commonly used cyclophotocoagulative technique [52, 53].

The contact treatment induces damage to the pigmented and nonpigmented epithelia and the stroma of ciliary processes, without a secondary effect to the overlying sclera [54]. The advantage of the contact procedure is to reduce the IOP using the same amount of energy than the that of the noncontact Nd:YAG procedure, but with an ability to deliver the energy sixty times lower, this leads to less tissue destruction and fewer postoperative complications. One of the most important studies on the efficacy of contact Nd:YAG cyclophotocoagulation was conducted by Schuman et al. (mean follow-up, 3.2 months) [55]. In this short-term follow-up study, 62% of patients reduced IOP under 22 mmHg and 49% under 19 mmHg. The preoperative IOP was 36.7 mmHg and decreased to 21.2 mmHg, with a mean IOP reduction of 15.5 mmHg; notably, the final IOP reduction was achieved soon after surgery, or within one week of treatment.

Afterwards, Brancato et al. used lower energy levels and fewer applications achieving similar results, even though IOP dropped under 20 mmHg in a limited number of cases [56]. In long-term follow-up studies conducted on contact cyclophotocoagulation in refractory glaucoma (2 to 10 years), the success rate of the procedure was reported to range from 37% to 92% [55, 57–64]. In these studies, mean preoperative IOP ranged from 29.9 to 40 mmHg and reduced from 15 to 21.8 mmHg. The most common postoperative complications described after contact Nd:YAG cyclophotocoagulation are reported in Table 2 [38, 65–69].

In 1992, Uram [70] reported the initial results of a novel ciliary body photocoagulation delivered under direct visualization through endoscopy, in patients with neovascular glaucoma. With respect to transscleral cyclophotocoagulation, which is reserved to intractable and advanced glaucoma, the endoscopic cyclophotocoagulation (ECP) is used also in nonrefractory cases, without absolute contraindications [71–73]. ECP has numerous advantages over transscleral cyclophotocoagulation, since the target tissue is directly visualized and, therefore, overtreatment is usually avoided.

Because of this, ECP was used in both mild POAG and advanced secondary glaucoma, also in combination with cataract surgery. In POAG, the IOP reduction was found to range from 18% to 47% (3.9 to 10.9 mmHg), with a mean IOP decrease of 31% (7 mmHg). In advanced secondary glaucoma, the IOP reduction ranged from 26% to 68% (7 to 28 mmHg) or yielded a mean IOP decrease of 50% (18 mmHg) [70, 71, 74–76]. In the largest retrospective study on ECP (7.4 years of follow-up), Lima et al. reported a postoperative IOP ranging between 6 and 21 mmHg in 79% of patients, with a mean number of medications of 1.9 [75].

Usually, ECP presents transient complications such as anterior chamber inflammation (22%), hyphema (11%), or cystoid macular edema (10%). The serious and potentially vision-threatening complications are less frequent in external cyclophotocoagulation and are represented by persistent hypotony (1–9%), phthisis (15 case reports), retinal detachment (1–6%), and vision loss or reduction (3–24%), especially in more advanced stages [71].

In closing, ECP is an effective and relatively safe procedure in recalcitrant glaucoma, which can be considered as a surgical option also in very selected cases of nonrefractive glaucoma.

## 4. High-Intensity Focused Ultrasounds (HIFU)

The HIFU technology, which is based on the favourable effects of high-frequency ultrasounds, is used in many fields of medicine. HIFU was initially proposed to treat different central nervous system diseases [77, 78]. Afterwards, in 1970s, its application was extended also in oncology, to induce a prolonged hyperthermia (elevation of tissue temperature to 43°C for one hour) in the entire tumor volume [79].

In ophthalmology, the HIFU technology was tested to treat retinal diseases, crystalline lens diseases, and choroid plexus diseases and to partially destroy the ciliary body. Baum and Greenwood showed that an ultrasound beam could disperse the ocular blood [80]; Purnell et al. published early results on cataract development and treatment of chorioretinal lesions [81]; Coleman et al. produced cataracts in rabbit lenses, observing the thermal mechanism underlying the final effect of high-intensity ultrasounds [11]. They also obtained the first in vivo threshold curves to induce chorioretinal lesions in albino rabbits, for the treatment of retinal detachment.

In the 1980s, the device was investigated for treatment of glaucoma. Coleman et al. conducted the first studies to evaluate the efficacy and safety of high-intensity focused ultrasounds (HIFU) in patients with uncontrolled IOP and advanced glaucoma [11, 12]. The strategy produced a commercially available device called as Sonocare Therapeutic Ultrasound System Model (Sonocare Inc., Ridgewood, New Jersey, USA) [82]. In the Sonocare system, the transducer was a single-spherical piezoceramic with 80 mm of diameter, working with a 4.6 MHz frequency. The system, which was attached to an articulated arm, required a 37° bath of saline solution to couple the eye with the transducer. The procedure was repeated to produce six pinpoint lesions of the ciliary body.

In the study of Coleman et al. at the third month of follow-up, IOP was less than 25 mmHg or 18 mmHg in 83% and 62% of patients, respectively [12]. In a larger case series, Burgess et al. reported similar results, reporting IOP values less than 25 mmHg in 90% of patients three months after the procedure [13]. At one year of follow-up, IOP was ≤25 mmHg in 65% of patients. The authors also documented the same efficacy of the procedure in retreating failing or unresponsive cases. Sterk et al. reported a 42.2% of IOP reduction after three months of follow-up in the 44 eyes with uncontrolled refractory glaucoma [15].

Several mechanisms of action were proposed to explain the final IOP lowering after HIFU, such as localized destruction of the ciliary-pigmented and nonpigmented epithelium, atrophy of the ciliary muscle, cyclodialysis cleft, and scleral thinning [11–13, 83]. Despite encouraging initial evidence, the ultrasound cyclodestruction was used only in advanced and refractory glaucoma, because of the significant risk of complications (scleral staphyloma, corneal thinning, persistent hypotony, phthisis bulbi, and loss of the visual acuity). Moreover, the particular complexity and duration of insonification with the Sonocare system led to progressively abandon the procedure in the middle of 1990s.

By refining the transducer design, the modes of energy delivery and the real-time imaging of the HIFU technology was rediscovered in oncology in the 1990s as an additional effective strategy to treat cancer. Currently, this technology is particularly used for primary solid tumors and metastatic diseases and to enhance the drug delivery through tissues. Uterine fibroids, prostate cancer, pancreatic cancer, liver tumors, and thyroid tumors are the main solid tumors accessible to the ultrasound energy benefit [84–87].

The availability of advanced imaging technologies such as the magnetic resonance thermometry and particular ultrasound imaging techniques permits the real-time monitoring of treatment effects induced by HIFU.

## 5. Miniaturized High-Intensity Focused Ultrasounds for Cyclodestruction in Glaucoma

In the last years, a miniaturized HIFU device assembled to precisely match with the ocular globe geometry was developed to insonify the ciliary body in uncontrolled refractory glaucoma. The device consists of a disposable therapeutic circular probe, a coupling cone, and a touch screen console; the coupling cone and the probe are connected to the console by means of a tube and an electric cable, respectively, and a foot pedal allows the activation of the treatment. The procedure was named as ultrasonic circular cyclocoagulation (UC^3^).

**Figure 1:**
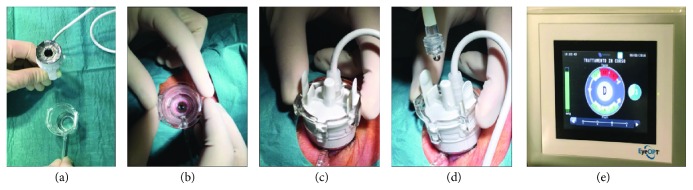
UCCC procedure. HIFU device (new-generation probe) comprises two elements: the probe with the six piezoelectric transducers generating the ultrasound beam and the coupling cone (a). The correctly positioned cone must show a homogeneous ring of visible sclera; when this ring is regular, the cone is then maintained by a mild vacuum system (b). After verification of the effective suction, the probe is inserted and stabilized into the cone (c). During the procedure, the cone is continuously filled with saline solution (d), in order to allow the ultrasound transmission. The treatment starts in the superior sectors with a progressive activation of each transducer (e).

The device (Figure 1) allows a sequential, computer-assisted treatment of the cylinder-shaped regions of the ciliary body, in a quick one-step circular procedure, thus eliminating the need to move the probe during the treatment. The circular shape of the probe, reproducing the macroscopic anatomy of the ciliary body, allows a high-precision coupling with the target organ (thus sparing the neighbouring structures) and permits a nonoperator-dependent treatment with highly reproducible lesions of the target organ [19]. To selectively impact with the ciliary body, the ultrasound beam is focused at a depth of 2 mm below the sclera, corresponding to the spatial position of the ciliary body.

In order to be safe and efficient, the system respects four anatomical constraints: (i) avoiding insonification of the cornea and the lens (obtained by a transducer aperture of 36°), (ii) avoiding the nasal and temporal zones during treatment, in order to preserve a sufficient aqueous humor production (the angle between the two transducers in the nasal and temporal sectors is 70°), (iii) minimizing the propagation distance through tissues, with the aim to reduce the attenuation of the energy, and (iv) avoiding a retinal overexposure (obtained by choosing a cylindrically shaped transducer).

The probe, which is 30 mm in diameter and a 15 mm high ring, is divided in six cylindrical piezoceramic transducers generating six ultrasound beams that allow treating up to 30% of the ciliary body. Transducers were operated at 21 MHz of frequency with an acoustic power of 2 watts; ultrasounds rapidly increase the local temperature of the ciliary body (up to 90° to avoid tissue boiling). The transducers are elliptic cylinder-shaped segments of a 10.2 mm radius, with a 4.5 mm width and a 7 mm length, generating an active surface area of 35 mm^2^. The result is a highly precise focusing of the target zone, not exceeding 0.1 mm × 1 mm in size. Transducers are equidistant between them, distributed three in the superior and three in the inferior regions. The focal volume of transducers presents an elliptic cylinder shape, which finally coagulates the same volume of the ciliary body.

The probe is inserted into a truncated polymer-made coupling cone and placed in direct contact with the eye; this allows the optimal positioning of the probe in terms of centering and distance and a stable alignment to the optical axis. The coupling cone is connected to a suction ring, which allows the application of a low-level vacuum to maintain the cone in contact with the ocular surface during the procedure, without movement and misalignment. A one dual-function foot pedal allows activating the suction and the firing phases directly by the surgeon or the second operator. Probes are commercialized in three different ring diameters (11, 12, and 13 mm), which allow them to fit most ocular sizes, except in cases of nanophthalmos or primary or secondary megalophthalmos. The probe size is determined before surgery by using ultrasound biomicroscopy (UBM), which permits to simulate the locations of the focal zones; the model that best targets the ciliary body is then chosen [16]. For UBM assessment, radial and transverse scans are obtained at 0°, 45°, 90°, 135°, 180°, 235°, 270°, and 315° meridians.

The main module of the HIFU device is constituted by the following components: (i) a signal generator producing an electrical voltage, (ii) an amplifier that enhances the electric voltage and allows transducers to be excited and produce ultrasounds, (iii) a watt meter that measures the incident and reflected electric power during the insonification, (iv) an electronic switch controller, which enables the electric voltage to be sent to transducers, and (v) a computer that controls the electronic switch and the signal generator and permits to set up the treatment parameters (frequency, power, duration, and number of sectors to treat). The computer sequentially activated sectors during treatment.

According to patient and physician preferences the procedure can be performed under topical, peribulbar, or general anaesthesia; nevertheless, anaesthesia is locally administered in most parts of cases.

### 5.1. UC^3^ Procedure

After registration of the surgeon name and the patient demographic data, the operator connects the probe to the console and selects the eye. The device automatically recognizes the probe and the suction test starts after clumping of the suction tube. In the next step, the surgeon puts in contact the coupling cone with the ocular surface and gently moves the cone to obtain a correct positioning and centering (a homogeneous white scleral ring surrounding the cornea should appear). The surgeon activates a 70 mmHg suction from the foot pedal, and when the optimal suction has been obtained (green bar on the screen), the probe is inserted into the cone and the position is maintained throughout the treatment. To facilitate the ultrasounds transmission, the cone is finally filled with balanced saline solution. At this stage, the device is ready to use and the treatment can start by selecting the firing button of the foot pedal. Transducers are sequentially activated clockwise, starting from the superior sectors both in the right and in the left eyes. Each transducer is activated for 4 or 6 seconds, with 20 seconds of interval between each sector, and the passage between sectors is completely automatic without the need to release the foot pedal. The particular interval between the activation of adjacent sectors allows the heat to be completely evacuated. The entire treatment, according to the selected regimen of insonification, lasts 2 minutes and 4 seconds (in the 4-second regimen) or 2 minutes and 16 seconds (in the 6-second regimen).

In the 4-second regimen, the volume of the destroyed ciliary process corresponds to 4.8 mm^3^, while in the 6-second regimen, 7.8 mm^3^; the regimen dose selection generally depends on the preoperative clinical status of the patient, in order to produce a lower or higher AH inflow reduction.

The console screen allows controlling the successful sequential activation of transducers during the whole procedure.

In the last year, a new-generation probe (Figures 1(a)–1(d)) with a modified coupling cone was commercialized, and it replaced the first-generation probe. The objectives of the technical modifications were to make the UC^3^ procedure even more intuitive and surgeon-friendly and to further boost clinical efficacy without compromising the favourable safety profile. The device was successfully redesigned to make the intraoperative handling simpler and smoother. The treatment probe was modified to treat up to an average 45% of the entire circumference of the ciliary body, increasing the active surface of the transducers from 2.5 to 4 mm in width. In this way, the active surface now covers almost the entire area of the transducer. Different from the original procedure, where the surgeon can select the desired time dose regimen (4 or 6 seconds), the novel probe offers a unique 8-second dose exposure for each of the six transducers, maintaining the same interval between sectors. Therefore, the procedure currently lasts 2 minutes and 32 seconds.

At the end of the treatment, patients receive topical antibiotics and steroids three times a day for 1 week, according to surgeon preferences and the postoperative course, and cyclopentolate twice daily for 4 days. In the first weeks after surgery, the IOP-lowering medications are generally maintained.

### 5.2. Efficacy and Safety of the Procedure

Clinical studies, conducted with the first-generation probe in patients with refractory glaucoma, reported encouraging results, especially in the early postoperative period. Mastropasqua et al. reported an overall success rate of 63.6% at month 1, with a higher success rate in the 6-second dose regimen (80%) compared to that in the 4-second dose regimen (41.6%) (UC^3^ was considered successful when at least a 30% reduction from preoperative IOP was obtained at one-month follow-up.) [88]. These results were in line with the 66.7% reported by Denis et al. in both groups of treatment at month 1 [17].

Considering the percentage IOP reduction, Mastropasqua et al. reported values of 30.1 and 38.7% in the 4-second and 6-second dose regimens, respectively, which were almost in line with literature that reported percentage reduction ranging from 22.8 to 26.4% in the 4-second regimen and from 28.2 to 38.2% in the 6-second regimen at month 1 [17]. The mild differences could probably depend on the different stages of disease and the typology of refractory glaucoma enrolled in the studies. The same studies produced partially conflicting results when considering longer follow-up. At 12 months, Aptel et al. reported an overall success rate of 83.3%; successful procedures were complete in 50% of cases and qualified in 68% of cases [16, 18]. Denis et al. reported a success rate ranging from 48% to 57% (Groups 2 and 1, respectively), and Melamed et al. reported a success rate of 65% [17, 21].

In these studies, preoperative IOP ranged from 27.5 to 39.1 mmHg, whereas postoperative IOP ranged from 17.1 to 23 mmHg at the last follow-up [16–18, 21, 88, 89].

Overall, based on these results, it seems that the procedure tends to maintain the IOP-lowering efficacy in the first year, with a success rate ranging from 48% to 83.3%, without reduction of the topical IOP-lowering medications.

The mean number of the UC^3^ procedure in the first 12 months ranged from 1.05 to 1.13 in the study of Aptel et al., while Denis et al. reported percentages of retreatment from 17.6 to 29.4% in the 6- and 4-second dose regimens, respectively [17, 89]. Finally, all these studies did not report a significant reduction of the mean number of medications after the procedure, especially in the long-term studies. On this basis, the UC^3^ usually produces a qualified surgical success. Though the new probes have been introduced to increase the efficacy of the procedure, to date, no direct comparative study has been published.

In all studies, the procedure was reported to be safe without serious intra- or postoperative complications. The most frequent complications were described in the early postoperative period (1 week) and were represented by conjunctival hyperaemia, punctate keratitis, subconjunctival hemorrhage, anterior chamber inflammation, and a transient IOP increase (more than 10 mmHg from baseline) (Table 3).

The introduction of the new probe allowed maintaining a high level of safety, even though in our initial case series, we noted a slight higher incidence of anterior chamber inflammation (cellularity and proteins determined by a slit lamp examination and graded according to the Likert scale).

A transient IOP increase occurs also during the procedure, because of the suction needed to couple the device with the ocular surface; this should be carefully considered in relation to the visual field of the treated eye. Though the occurrence of optic nerve and retinal vascular changes after UC^3^ has been not documented, studies on subjects undergoing LASIK (that similarly requires suction) reported cases of optic neuropathy and visual field loss related to the suction process [90, 91]. Considering these potential complications, UC^3^ was not recommended in advanced/end-stage glaucoma.

Serious complications such as persistent hypotony or phthisis, which were relatively common in other cycloablative techniques (occurring 6 to 30 months after surgery), were never described after UC^3^. Given the high rate of safety demonstrated in refractory glaucoma [16–21, 88], Aptel et al. recently conducted a study to evaluate the efficacy and safety of UC^3^ in patients with early glaucoma, naïve of any previous filtering surgery [89]. The authors reported a complete and qualified success of 46.7% and 63%, respectively, with a mean IOP reduction of 37% at 12 months, using the 6-second regimen.

Based on these encouraging results, the indication for the procedure has been extended also to patients with primary or secondary open-angle glaucoma naïve to filtration surgery.

### 5.3. Mechanisms of Action of UC^3^

The AH inflow reduction following the thermic necrosis of the ciliary epithelium seems to play the main role in the final IOP lowering after UC^3^. In the pilot histopathology studies conducted on rabbits, Aptel et al. found that the distal and intermediate parts of the ciliary processes presented necrotic changes of the stroma and epithelium, ranging from oedema to vascular congestion; conversely, the basal parts of the ciliary processes and the rest of the ciliary body appeared normal [19, 92]. Focal interruptions and disruptions of the ciliary processes and pars plana microvasculature were also observed with light and scanning electron microscopy.

On the other hand, the evenly delivered ultrasound dose did not induce significant inflammatory reactions in the treated portions and permitted a good preservation of the blood aqueous barrier. The adjacent untreated areas presented normal ciliary epithelium and stroma, no signs of inflammation, and a complete preservation of the 3D vasculature. These anatomical aspects confirm that high-frequency ultrasounds are precisely focused on the target volume, producing histological lesions strictly limited to the site of sonication. These findings also represent the basis for the higher clinical safety of UC^3^ compared to those of standard cyclodestructive procedures, which do not spare the neighbouring tissues during the energy delivery.

Besides the effects on the ciliary body, an increase of the suprachoroidal and transscleral AH outflow has been also documented [16–19]. A hypoechogenic suprachoroidal space was observed in 67% patients one month after the ciliary body insonification; this indicated an increased uveoscleral outflow through the supraciliary and suprachoroidal space, in line with histological findings.

**Figure 2:**
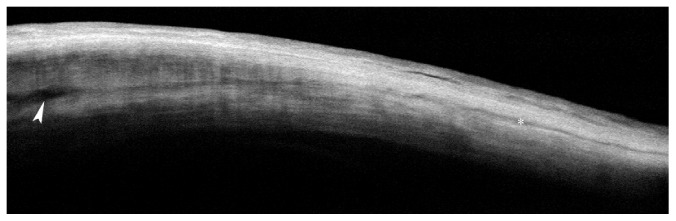
Anterior segment-optical coherence tomography of the sclera before insonification. Preoperative normal sclera presenting a relatively homogeneous stroma, with some scattered linear- (asterisk) or oval- (arrowhead) shaped hyporeflective spaces interspersed between the collagen fibres.

**Figure 3:**
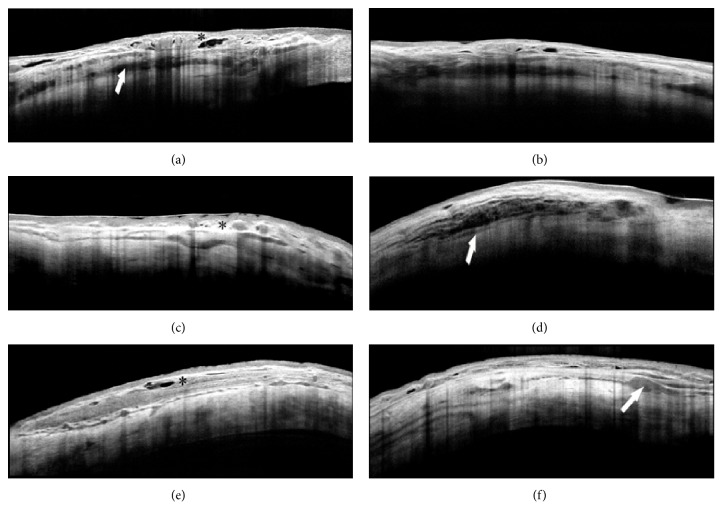
Anterior segment optical coherence tomography of scleral modifications after successful UCCC. (a–c) Second-generation probes (8-second treatment; 4 mm wide active area); (d–f) first-generation probes (6-second treatment; 2.5 mm wide active area). Intrascleral hyporeflective spaces (arrows and asterisks), with a different degree of internal reflectivity, are clearly recognizable within the stroma. These spaces are prominent after seven days from the treatment (a), and persist, even though reduced, after one (b) and three (c) months. No significant macroscopic differences are detectable between the two generation probes, even though the current probes seem to induce a greater scleral delamination. Scans were taken at the superior-temporal quadrants, 3 mm from the site of previous filtration surgery.

**Figure 4:**
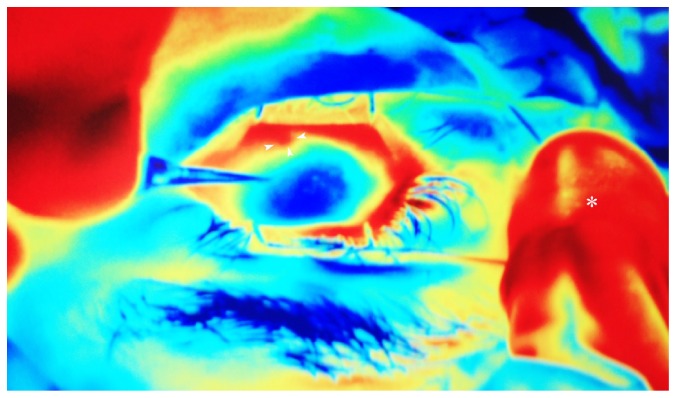
The image shows an ocular surface thermogram obtained with a digital infrared camera, of a representative patient during 6-second dose insonification, immediately after removal of the 6 o'clock hour transducer. A single evident circular red spot is well distinguishable (arrowheads), which corresponds to an area of increased temperature at the site of transducer. Asterisk indicates the nose of the patient.

In a recent study, our group observed significant modifications of the scleral and conjunctival anatomy one month after UC^3^, in patients insonified with either 4- or 6-second regimen [88]. Anterior segment optical coherence tomography documented the formation of new (or the enlargement of preexisting) intrascleral hyporeflective spaces (HSs) 1 month after the procedure. HSs were defined as intrastromal cavities presenting a lower degree of reflectivity compared to those of the surrounding sclera. Intrascleral HSs markedly increased from two to three times with respect to baseline (Figures 2 and 3) only at the site of transducer contact, without involvement of the surrounding sclera. We hypothesized that the HS increase was a consequence of a thermic-induced scleral fibre delamination; in fact, a heating of the suprachoroid, sclera, and conjunctiva during the procedure may occur, given that the transducer produces a thermic halo (1.89 mm^3^) with a temperature gradient from the ciliary body to the ocular surface. The preliminary results of an ongoing thermal infrared imaging study seem to support this hypothesis, since we observed a significant increase in the ocular surface temperature at the site of insonification, immediately after the UC^3^ (Figure 4). This thermic effect may also account for the higher HS increase in patients treated with the 6-second dose regimen, which received a prolonged duration of the insonification. The increase of such HSs leads to an enhancement of the scleral hydraulic conductivity and, therefore, of the AH transscleral outflow.

**Figure 5:**
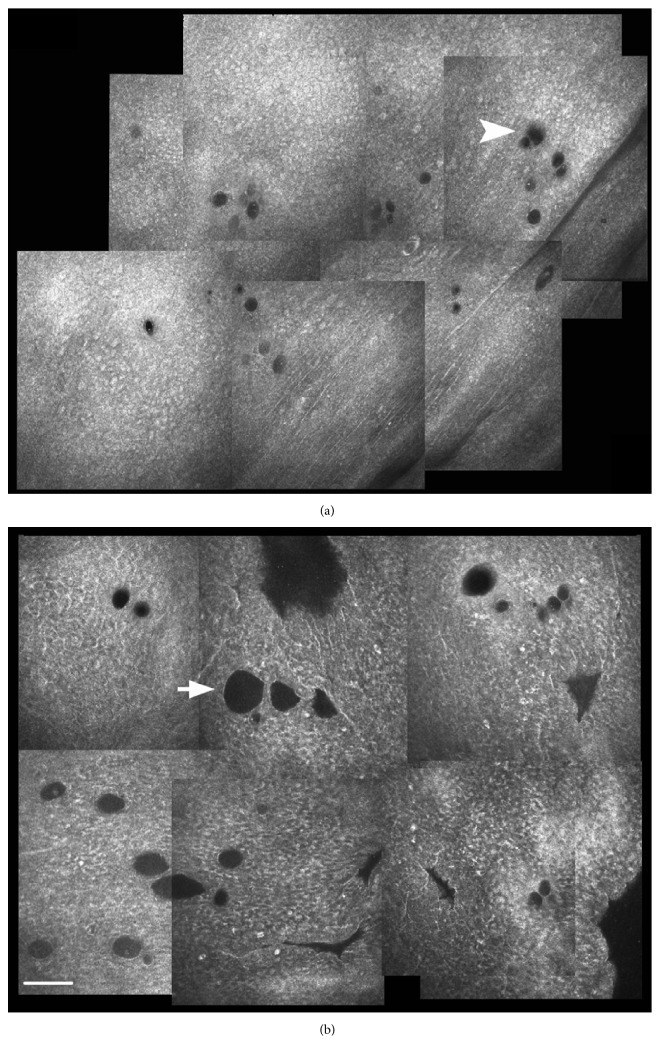
In vivo confocal microscopy of the superior temporal conjunctiva in the same patient scheduled to undergo a 4-second dose UCCC (Group 1). (a) The baseline planar reconstruction shows small roundish microcysts, located at different levels within the epithelium, scattered, and sometimes clustered (arrowhead). (b) Microcysts increased density and area (arrow) thirty days after insonification. Bar represents 100 *μ*m (from [88], with permission of the publisher).

**Figure 6:**
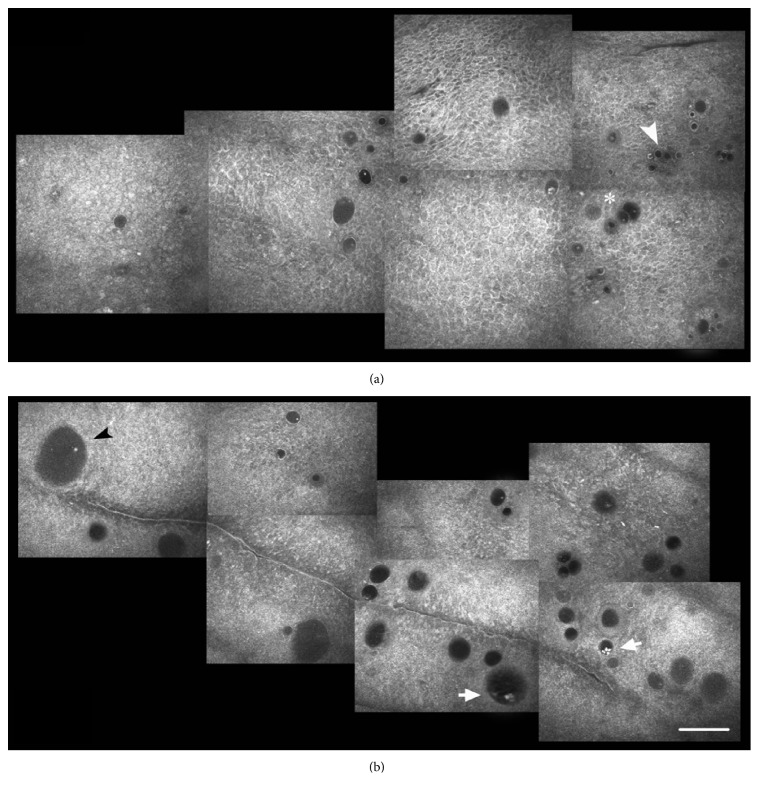
In vivo confocal microscopy of the superior temporal conjunctiva in the same patient scheduled to undergo a 6-second dose UCCC. (a) The baseline planar reconstruction shows features similar to those observed in Group 1. Somewhere, microcysts appear encapsulated (arrowhead) and filled with amorphous material or punctate reflective elements (asterisk). (b) Epithelial microcysts increased density and, especially, area (arrow) thirty days after UCCC. Microcysts may appear filled with amorphous material (black arrowhead) or reflective elements, probably representing inflammatory cells (arrows). Bar represents 100 *μ*m (from [88], with permission of the publisher).

In vivo confocal microscopy confirmed the transscleral outflow enhancement one month after the procedure by documenting a significant increase of conjunctival microcysts at site of insonification (Figures 5 and 6). These microcysts were proposed as an in vivo hallmark of the AH passage through the sclera and finally the conjunctiva [93–100].

The scleral architecture remodelling observed after UC^3^ may pose concerns in patients candidate to further filtration surgery, since the (intra- and postoperative) resistance of the sclera and the AH permeability of collagen fibres could be significantly altered, especially after repeated sonications. At this moment, there are no studies that addressed this point; therefore, these aspects must be considered and carefully pondered before proposing filtration surgery after HIFU, either in refractory or (even more) in nonrefractory cases. In addition, there are no long-term studies that evaluated the risk of hypotony after repeated insonifications or the risk of hypotony whether patients receive further filtration surgery. Despite no comparative randomized clinical trials have been performed, an overview of literature leads to a hypothesis that UC^3^ might have a little lower efficacy (also in terms of reduction of number of medications) and provide a shorter duration of the IOP-lowering effect compared to that of the other cyclodestructive approaches, though with a greater safety profile [16–19, 42, 43, 54–69, 88]. On the other hand, the new probes seem to increase the IOP-lowering efficacy of the technique, maintaining the same level of safety.

In closing, even though promising and safe, the ultrasonic cyclocoagulation still requires correct positioning in terms of indication and timing, in the management of glaucoma.

## 6. Summary and Conclusions

Currently, cyclodestructive procedures are exclusively limited to refractory/end-stage glaucoma, because of the high incidence of vision-threatening complications. All proposed procedures are noncompletely selective for the target organ, have an unpredictable dose-effect relationship, are operator dependent, and are poorly reproducible. UC^3^ is an emerging and encouraging technique, which utilizes the HIFU technology to induce a one-step, automated, computer-assisted, non-operator-dependent, and highly reproducible thermal coagulation of the ciliary epithelium. This procedure allows a selective destruction of the limited and predefined portions of the ciliary body, thus reducing the AH inflow in a controlled way. UC^3^ presents several advantages over traditional cyclodestructive techniques since it minimizes the intra- and postoperative complications, preserves neighbouring organs from undesired treatment, allows a faster postoperative recovery, and permits retreatments (by rotating transducers) because there is no dose limit.

Besides the reduction of the AH inflow, which is the main mechanism that reduces IOP, UC^3^ increases also the AH outflow, by favourably remodelling the anatomical architecture of suprachoroid, sclera, and conjunctiva. This indicates that UC^3^ may influence the entire hydrodynamic system, exploiting different mechanisms to finally reduce the IOP. The promising results, along with the high level of safety reported in refractory glaucoma, allowed extending the indication for UC^3^ also in glaucomatous patients naïve to surgery, thus reconsidering the role and the timing of cyclodestruction in the management of glaucoma.

However, to date, no comparative study between UC^3^ and other cyclodestructive procedures has been published. Therefore, whether UC^3^ represents a better solution for refractory glaucoma with respect to standardized cycloablative techniques needs to be addressed.

The described effects of high-frequency ultrasounds on the sclera and conjunctiva might open future strategies to lower IOP in glaucoma. In fact, the development of modified HIFU probes that will focus the ultrasonic beam just within the sclera avoiding the ciliary body could stimulate the uveoscleral outflow pathway by increasing the transscleral AH resorption. This may have the great advantages to reduce the IOP by stimulating the physiological AH outflow routes and reduce the postoperative complications by preserving the ciliary body, which plays a critical role in the global health of the eye.

## Figures and Tables

**Table 1:** Complications after cyclocryotherapy.

Complications		Incidence	Reference
Mild	AC flare/uveitis	17.6%–100%	[3], [28], [32], [40]
	Hyphema	4%–17.6%	[4], [29], [30], [40]
	Sterile hypopyon	1.5%	[28]
	Lens dislocation	1 case reported	[31]

Severe/vision threatening	VA loss	5.3%–58%	[3], [28], [29], [32], [36]
	VA decrease^∗^	32.3–45.1%	[28], [32]
	Hypotony	3.33%–32%	[33–35]
	Phthisis bulbi	3.3%–40%	[3], [29], [32], [37], [40]
	Choroidal detachment	2%	[3], [4]
	Retinal detachment	1.6%	[3], [29]
	Sympathetic ophthalmia	?	[41]

VA: visual acuity; ?: unsolved question.

^∗^≥2 Snellen lines.

**Table 2:** Complications after transscleral contact cyclophotocoagulation.

Complications		Incidence	Reference
Mild	AC flare/uveitis	9%–28%	[57], [58], [64], [65]
	Hyphema	0%–2%	[55], [58], [59]
	Pain	9%–21%	[55], [58], [64]
	Pupillary changes	0.8%–50%	[57], [61]

Severe/vision threatening	VA loss	8.8%–47%	[38], [54], [56], [64], [68]
	VA decrease^∗^	38.5%–62.5%	[56], [57], [59]
	Hypotony	0%–26%	[3], [43], [56], [58], [61], [66]
	Phthisis bulbi	0%–10.7%	[39], [54], [55], [61], [67]
	Retinal detachment	1%	[55]
	Sympathetic ophthalmia	?	[3], [41]

VA: visual acuity; ?: unsolved question.

^∗^≥2 Snellen lines.

**Table 3:** Complications after UC^3^.

Complications		Incidence
Mild	Intraoperative pain	4.1%–10.7%
	Conjunctival hyperaemia	37.5%–75%
	Subconjunctival hemorrhage	4%–16.6%
	Superficial punctate keratitis	10.7%–40%
	Anterior chamber reaction	16.6%–40%
	IOP spikes^∗^	6.6%–20.8%
	Focal scleral thinning	3.3%

Severe/vision threatening	Transient VA decrease^∗∗^	10.7%
	Transient hypotony	1.6%–5%
	Corneal edema	7.1%–8.3%
	Corneal ulceration^§^	8.3%–16.6%
	Transient macular edema	3.3%–3.6%

UC^3^: ultrasonic circular cyclocoagulation; VA: visual acuity.

^∗^IOP increase higher than 5 versus baseline, in the first week.

^∗∗^≥2 Snellen lines, transient.

^§^Patients with preexisting corneal disorders.

References [16–21, 88, 89].
